# Towards Precision Medicine in Metastatic Renal Cell Carcinoma: The Role of Emerging Biomarkers

**DOI:** 10.3390/cancers18081228

**Published:** 2026-04-13

**Authors:** Rugile Pikturniene, Alvydas Cesas, Sonata Jarmalaite, Edita Baltruskeviciene, Vincas Urbonas

**Affiliations:** 1 Life Sciences Center, Institute of Biosciences, Vilnius University, 01513 Vilnius, Lithuania; 2 Oncology Department, Klaipeda University Hospital, 92288 Klaipeda, Lithuania; 3 Oncology Department, Vilnius University Hospital Santaros Klinikos, 08661 Vilnius, Lithuaniavincas.urbonas@nvc.santa.lt (V.U.)

**Keywords:** metastatic renal cell carcinoma, precision oncology, biomarkers, predictive biomarkers, immune checkpoint inhibitors, tumour microenvironment, circulating tumour DNA

## Abstract

RCC remains a therapeutically challenging malignancy, particularly in its metastatic stage, in which treatment resistance and limited response durability persist despite recent advances in immunotherapy and targeted therapies. Although immune checkpoint inhibitors (ICIs) have significantly improved outcomes for a subset of patients, reliable prognostic and predictive biomarkers to guide therapy selection are still lacking. Current clinical models, such as the International Metastatic RCC Database Consortium (IMDC) risk score, offer only limited insight into the molecular and immunologic complexity of RCC. Emerging molecular biomarkers implicated in resistance mechanisms reflect the underlying heterogeneity of RCC and may inform future therapeutic strategies. Kidney Injury Molecule-1 (KIM-1), a transmembrane protein that is up-regulated in RCC and detectable in circulation, has demonstrated potential as a non-invasive biomarker for diagnosis, prognosis, and treatment monitoring. Liquid-biopsy approaches, including the analysis of circulating tumour DNA (ctDNA), microRNAs (miRNAs), and long non-coding RNAs (lncRNAs), are also gaining traction due to their minimally invasive nature and potential for real-time disease monitoring. This review aims to provide a structured overview of emerging biomarkers in metastatic RCC, critically evaluate their current clinical applicability, and propose a biologically informed framework for their integration into clinical decision-making. In addition, we propose a conceptual IMDC-Plus framework that integrates clinical, biological, and early dynamic markers to improve risk stratification in the era of immunotherapy (IO).

## Simple Summary

Renal cell carcinoma (RCC) is a complex disease that remains challenging to treat, particularly in the metastatic setting. Although recent advances, including immunotherapy, have improved outcomes, it is still difficult to predict which patients will benefit most from specific treatments. This highlights the need for reliable biomarkers that can support more-informed clinical decision-making. In this review, we summarise the most promising emerging biomarkers detectable in blood and tumour tissue and discuss their potential role in clinical practice. We also describe a conceptual approach that combines clinical features with biological and early treatment-related changes to better reflect disease behaviour. Such strategies may help move towards more-personalised treatment in renal cell carcinoma.

## 1. Introduction

The global incidence of RCC continues to rise, while earlier detection through increased use of imaging has led to a higher proportion of cases being diagnosed at earlier stages. Although the broader use of diagnostic computed tomography (CT) imaging has increased the detection of early-stage disease, approximately one-quarter of patients are still diagnosed with disseminated cancer. Likewise, nearly one-quarter of those who undergo surgery with curative intent eventually experience progression to metastatic disease [1,2,3,4,5].

RCC comprises a diverse spectrum of malignancies defined by distinct histopathological subtypes and intricate molecular features. The therapeutic landscape for metastatic RCC (mRCC) has become increasingly complex as the range of available treatment regimens expands. Surgical resection remains the primary therapeutic approach for localised RCC (lRCC); however, in cases of advanced or disseminated disease, immunotherapy (IO)—either alone or in combination with a tyrosine kinase inhibitor (TKI)—represents the current standard of care [6,7].

In daily clinical practice, management is based on patient age, performance status, comorbidities, and routine biochemical markers such as lactate dehydrogenase (LDH) and C-reactive protein (CRP), alongside IMDC criteria. These factors guide treatment decisions; however, the ability to select truly individualised therapy remains limited. This review discusses the need for reliable biomarkers, summarises the current evidence, and outlines future perspectives regarding their integration into routine clinical care.

## 2. Materials and Methods 

A structured literature search was performed to identify relevant studies on biomarkers in renal cell carcinoma. Electronic databases including PubMed, Web of Science, and Scopus were searched for articles published between 2010 and 2025. The search strategy included combinations of keywords such as “renal cell carcinoma”, “biomarkers”, “immunotherapy”, “circulating tumor DNA”, “circulating tumor cells”, “microRNA”, “non-coding RNA”, and “kidney injury molecule-1”. Studies were selected based on relevance to the topic, with priority given to recent high-quality clinical trials, translational research, and comprehensive review articles. Both retrospective and prospective studies were considered. Articles not published in English, case reports, and studies lacking sufficient methodological detail were excluded. Given the narrative nature of this review, formal systematic review methods were not applied; however, efforts were made to ensure a balanced and comprehensive representation of the current evidence.

## 3. Risk Models and Current Biomarkers

### 3.1. IMDC

The IMDC risk model remains the only prognostic tool that has undergone prospective validation in a phase 3 randomised controlled trial. Initially designed for metastatic RCC, the IMDC model incorporates six clinical and routine blood biomarkers and has been validated across various patient populations—including those with non-clear-cell and papillary RCC, in later lines of therapy and in individuals treated with ICIs [8].

Developed in 2009 from multi-institutional analyses of 645 patients treated with agents such as sunitinib, sorafenib, or bevacizumab plus interferon, the model was created in the vascular endothelial growth factor (VEGF)-targeted therapy era and is now incorporated into clinical practice guidelines for mRCC management. It uses specific clinical and laboratory criteria (summarised in Table 1) to classify patients into favourable-, intermediate-, or poor-risk groups [9,10].

The IMDC risk stratification system may reflect distinct features of the tumour microenvironment. Tumours in the favourable-risk group often display an angiogenesis-driven phenotype, while those in the poor-risk category are more likely to exhibit immunogenic features. This biological divergence helps explain differences in response to systemic therapies: patients with favourable-risk RCC may respond more effectively to anti-angiogenic agents because of increased vascular signalling, whereas intermediate- and poor-risk groups typically harbour immune-evasive phenotypes, making them better candidates for ICI therapy [11] (Table 2). In addition, histological subtype is increasingly incorporated into clinical trial stratification, as biomarker development and treatment response differ substantially between clear cell RCC (ccRCC) and non-clear cell RCC (non-ccRCC).

### 3.2. Histologic Subtypes

Histologic classification plays a crucial role in prognostication and is increasingly relevant for treatment selection. While ccRCC remains the most prevalent subtype, non-clear-cell variants such as papillary, chromophobe, and collecting-duct carcinoma are more heterogeneous and often have poorer outcomes. Retrospective analyses show inferior survival among patients with non-clear-cell histologies compared with ccRCC, although the IMDC model retains prognostic value in these groups [12].

Particularly aggressive phenotypes such as sarcomatoid and rhabdoid RCC—arising from any histologic background—are marked by poor clinical outcomes and resistance to VEGF-targeted therapies. These variants often harbour distinct molecular features, including BRCA1-associated protein 1 (*BAP1*) or cyclin-dependent kinase inhibitor 2A (*CDKN2A)* mutations and increased MYC proto-oncogene (*MYC*) transcriptional activity [13]. Despite historically poor outcomes, these tumours show remarkable sensitivity to ICIs: in subgroup analyses from the CheckMate 214 trial, patients with sarcomatoid features demonstrated significantly improved overall and progression-free survival and higher response rates when treated with nivolumab plus ipilimumab compared with sunitinib [1].

Transcriptomic studies reveal that sarcomatoid tumours exhibit an inflamed immune microenvironment, with increased infiltration of CD8^+^ T cells, Th1 cells, and M1 macrophages and up-regulation of antigen-presentation machinery [14]. These features likely underlie the robust responses observed in clinical trials and real-world settings [15]. For other non-clear-cell subtypes such as papillary RCC, early-phase trials combining ICIs with VEGF inhibitors (e.g., nivolumab plus cabozantinib, pembrolizumab plus lenvatinib) show promising efficacy [16,17]. While these findings suggest potential benefit, evidence remains limited by small patient numbers and histologic diversity. Ultimately, histology provides valuable prognostic insight but is insufficient as a standalone biomarker for optimal treatment selection. Integration of genomic, transcriptomic, and immune-related markers is required to better stratify patients and personalise therapy in both clear-cell and non-clear-cell RCC [18].

### 3.3. Inflammatory Blood-Based Biomarkers

Systemic inflammatory markers derived from routine blood tests have gained attention as accessible, cost-effective prognostic indicators. These markers can be broadly grouped into systemic inflammatory indices and circulating cytokines, both of which reflect tumour–host interactions. Among the most widely studied are LDH, CRP, the neutrophil-to-lymphocyte ratio (NLR), and the platelet-to-lymphocyte ratio (PLR). Although non-specific, these biomarkers provide insight into tumour biology and host immune response, particularly when interpreted alongside other clinical parameters.

LDH plays a key role in anaerobic glycolysis and is released during cellular damage, hypoxia, or tumour necrosis—features common in rapidly growing malignancies. In mRCC, elevated LDH consistently associates with shorter overall survival. Its prognostic role has been confirmed in patients treated with VEGF-targeted therapies and is reflected in the IMDC risk model [19,20].

CRP, a hepatic acute-phase reactant primarily driven by interleukin-6, correlates with tumour burden and inferior outcomes. CRP has been combined with other parameters—such as albumin and lymphocyte count—to form composite scores like the C-reactive protein–albumin–lymphocyte (CALLY) index, which may improve risk stratification [21].

NLR, the ratio between circulating neutrophils and lymphocytes, is a dynamic marker of immune dysregulation. An elevated NLR reflects both pro-tumour inflammatory activity and suppression of cell-mediated immunity. A large meta-analysis encompassing over 6000 RCC patients found high baseline NLR strongly correlated with worse overall, progression-free, and cancer-specific survival [22]. However, most of the available evidence is derived from retrospective analyses, with variability in cut-off values across studies.

PLR has also been evaluated as a prognostic tool, though its predictive value appears less robust. While elevated PLR has been associated with unfavourable outcomes in some studies, inconsistencies in cut-off definitions and weaker associations compared with NLR have limited routine use [22].

Despite their convenience and low cost, these markers lack specificity and are easily influenced by comorbid conditions (e.g., infections, chronic inflammatory states), making them unreliable as standalone prognostic tools. Nevertheless, when integrated into validated models such as the IMDC criteria, they provide useful context for therapeutic decision-making and patient counselling.

In addition to routinely used inflammatory parameters, circulating cytokines provide a more direct reflection of tumour–host immune interactions in metastatic renal cell carcinoma. Among these, interleukin-6 (IL-6), interleukin-8 (IL-8), and interleukin-10 (IL-10) have been most consistently linked to adverse outcomes [23]. IL-6 plays a central role in cancer-related inflammation by promoting tumour growth, angiogenesis, and immune suppression, and elevated IL-6 levels are associated with increased tumour burden and inferior survival [24]. IL-8, a pro-angiogenic chemokine involved in neutrophil recruitment, has emerged as a marker of aggressive disease and reduced benefit from immune checkpoint inhibitors, particularly when circulating levels remain elevated during treatment [23]. IL-10 contributes to immune evasion by suppressing antigen presentation and T-cell activation, although its prognostic value in RCC appears less consistent compared with IL-6 and IL-8 [24]. However, the current evidence is largely derived from small and heterogeneous studies, and cytokine assays remain insufficiently standardised for routine clinical use. While cytokine measurements are not part of routine clinical practice, IL-6 and IL-8 in particular provide biologically meaningful information that may complement standard blood-based inflammatory markers when integrated into multi-parameter prognostic models such as the proposed IMDC-Plus framework [23,24].

## 4. Commonly Altered Genes in RCC

RCC is molecularly heterogeneous. In clear-cell RCC, loss of function of the von Hippel–Lindau (VHL) tumour suppressor is the most prevalent early event, stabilising hypoxia-inducible factors (especially hypoxia-inducible factor 2 alpha (HIF-2α)) and promoting transcription of genes involved in angiogenesis, metabolism, and proliferation (e.g., *VEGF*, platelet-derived growth factor (*PDGF*), transforming growth factor alpha (*TGF-α*)) [25]. On chromosome 3p—often co-deleted with *VHL*—tumour suppressors polybromo 1 (*PBRM1*), SET domain containing 2 (*SETD2*), and *BAP1* are recurrently mutated. *PBRM1* mutations (up to ~40%) involve SWItch/sucrose non-fermentable (SWI/SNF) chromatin remodelling and are often associated with a more indolent course [26]. *BAP1* mutations (~10–15%) correlate with high grade, aggressive behaviour, and worse overall survival (OS) [27]. *SETD2* loss (~10–12%) impairs DNA repair, promotes genomic instability, and may reduce sensitivity to ICIs and VEGF-targeted therapy [28].

These mutations may also shape the immune microenvironment. PBRM1 loss has been linked to enhanced interferon signalling and an increased likelihood of benefit from immune checkpoint inhibitors [29]. In contrast, BAP1 alterations have been associated with more-aggressive tumour behaviour and adverse clinical outcomes [27]. Non-clear-cell subtypes display distinct drivers—mesenchymal–epithelial transition (*MET*) alterations in type 1 papillary RCC and mitochondrial/mammalian target of rapamycin (mTOR) pathway alterations in chromophobe RCC—supporting subtype-specific strategies [30,31]. Although mutational profiling holds promise for informing prognosis and guiding therapy, clinical integration is limited by intra-/inter-tumour heterogeneity, lack of standardised testing, and the need for prospective biomarker-driven trials.

VHL inactivation activates hypoxia signalling; convergent evidence highlights HIF-2α as a key oncogenic driver in ccRCC [32,33,34]. This has led to development of HIF-2α inhibitors (e.g., belzutifan), with clinical activity in VHL-associated RCC and emerging roles beyond that setting [35,36]. The VEGF pathway remains central to mRCC therapy; VEGF-targeting tyrosine kinase inhibitors (TKIs) (e.g., sunitinib, axitinib, cabozantinib) are widely used, often in combination with ICIs [37,38]. Resistance is multifactorial: activation of alternative pro-angiogenic pathways, including fibroblast growth factor (FGF) and angiopoietin/TIE2 signalling (ANGPT/TIE2), as well as vessel maturation and pericyte stabilisation, and hypoxia-induced immunosuppression. Notably, VEGF blockade can intensify hypoxia, further stabilising HIFs and driving adaptation [8,39,40]. Targeting these mechanisms—especially with HIF-2α inhibitors—and optimising combinations/sequencing with ICIs may improve long-term outcomes, contingent on biomarker-guided patient selection.

## 5. Next-Generation Biomarkers

Liquid-biopsy approaches in RCC can be broadly categorised into three complementary components: circulating tumour DNA, circulating tumour cells and circulating non-coding RNAs, each providing distinct but overlapping insights into tumour biology.

### 5.1. Circulating Tumour DNA in Renal Cell Carcinoma

ctDNA consists of small fragments of tumour-derived DNA released into the bloodstream through apoptosis, necrosis, or active secretion. In oncology, ctDNA has emerged as a promising non-invasive biomarker with potential applications in early detection, prognosis, molecular profiling, and treatment monitoring. While ctDNA is widely studied in other solid tumours (e.g., lung and colorectal cancer), its clinical utility in RCC remains under active investigation due to biological and technical challenges unique to this disease.

RCC is characterised by relatively low circulating ctDNA levels, partly due to moderate tumour burden, vascular architecture, and less frequent mutations in canonical oncogenes compared with other cancers. Nevertheless, studies have demonstrated ctDNA detectability in metastatic RCC (mRCC), and its quantitative and qualitative features may carry clinically relevant information [40,41]. Specific mutations in genes—including *VHL*, *PBRM1*, *SETD2*, *BAP1*, and *MTOR*—can be reliably identified in ctDNA, reflecting the underlying tumour genomic profile [42]. Additionally, fragmentomic features (e.g., shorter fragment sizes and altered end motifs) have been proposed as biomarkers of ctDNA origin and disease dynamics [43].

ctDNA levels correlate with tumour burden and disease progression. Higher baseline ctDNA concentrations have been associated with shorter progression-free survival (PFS), whereas on-treatment decreases correlate with response [44]. ctDNA can also capture tumour heterogeneity more comprehensively than single-site tissue biopsy, which is particularly valuable in RCC, in which spatial and temporal heterogeneity is common [45].

Despite this promise, routine implementation in management of RCC faces technical limitations: low ctDNA abundance (especially in localised disease), lack of RCC-specific validated panels, and challenges distinguishing ctDNA from background cell-free DNA (cfDNA). Highly sensitive approaches—such as ultra-deep sequencing, methylation profiling, and fragmentomics—are being evaluated to overcome these barriers [41,43,46]. From a clinical perspective, ctDNA detection rates in RCC vary substantially by disease stage, with higher detection rates observed in metastatic disease and limited sensitivity in localised tumours. Mutation-based ctDNA panels have shown relatively low detection rates, likely reflecting low levels of tumour DNA shedding, whereas emerging approaches such as methylation profiling and fragmentomics appear more promising for improving sensitivity. Taken together, ctDNA represents a minimally invasive avenue for real-time molecular monitoring in RCC. Although not yet standard in clinical practice, ongoing prospective trials are likely to clarify its role in disease stratification, early relapse detection, and therapy guidance, particularly in the metastatic setting; however, most available data are derived from retrospective studies with relatively small cohorts, and prospective validation in RCC remains limited.

### 5.2. Circulating Tumour Cells

Circulating tumour cells (CTCs) are cancer-derived cells that detach from primary or metastatic lesions and enter the bloodstream, potentially serving as intermediaries in the metastatic cascade. Detection rates of CTCs in RCC are generally lower than in other epithelial malignancies, partly due to reduced expression of epithelial markers such as epithelial cell adhesion molecule. Although CTC analysis is being explored as a prognostic and predictive tool across multiple malignancies, its application in RCC remains under development, partly due to technical limitations and RCC’s biological features.

Unlike many epithelial cancers, RCC frequently exhibits low expression of traditional epithelial markers such as epithelial cell adhesion molecule (EpCAM), reducing the efficacy of conventional immunomagnetic capture methods [8,41]. Emerging technologies—microfluidic enrichment, size-based filtration, and marker-independent approaches—have improved detection sensitivity and enabled CTC identification even in advanced RCC [46].

Several studies suggest that CTC presence and quantity correlate with adverse clinical outcomes in mRCC. Increased CTC counts have been associated with poorer OS and PFS, positioning CTCs as dynamic biomarkers of disease burden and treatment response [47]. Fluctuations in CTC levels during therapy may reflect efficacy or resistance, offering a minimally invasive approach for longitudinal monitoring.

Beyond enumeration, molecular characterisation of CTCs provides additional insight. Unlike ctDNA, CTCs retain cellular structure, enabling transcriptomic, proteomic, and functional analyses. Profiling has revealed expression of resistance-associated genes (e.g., mesenchymal–epithelial transition (*MET*), *VEGFR2*, programmed death-ligand 1 (*PD-L1*)) that may be absent in archival tumour tissue or ctDNA samples [48], suggesting CTCs can act as a real-time proxy for tumour heterogeneity under therapeutic pressure. Preliminary data also indicate that PD-L1 expression on CTCs may correlate with ICI response, though this requires validation in larger cohorts [43].

Significant challenges remain: CTCs are rare (often <1 cell/mL), and the lack of standardised detection/characterisation protocols limits cross-study comparisons. Most data are from small, retrospective series, underscoring the need for prospective validation. In conclusion, CTCs represent a promising liquid-biopsy modality for prognosis, therapeutic stratification, and treatment monitoring in RCC, although the current evidence is largely based on small, heterogeneous studies, and standardised detection methods are lacking.

### 5.3. microRNAs and Long Non-Coding RNAs

Non-coding RNAs—particularly miRNAs and lncRNAs—are increasingly investigated in RCC as potential biomarkers due to their regulatory roles in gene expression and involvement in key oncogenic pathways. Unlike traditional tissue-based markers, these molecules can often be detected in circulation, offering a minimally invasive means of assessing tumour biology.

Among the most studied miRNAs is miR-210, a “hypoxamir” induced by HIF-1α. Elevated miR-210 consistently associates with hypoxic tumour environments and correlates with aggressiveness and unfavourable prognosis [49,50]. Goto et al. reported that high miR-210 levels were associated with shorter OS in clear-cell RCC, suggesting a role in risk stratification [49]. miR-1233 has diagnostic potential: serum levels are significantly higher in RCC than in healthy controls, a finding supported by meta-analytic data confirming the diagnostic relevance of both miR-210 and miR-1233 [51].

Among lncRNAs, metastasis-associated lung adenocarcinoma transcript 1 (MALAT1) has been linked to migration, invasion, and metastasis, with higher plasma levels associated with poorer outcomes [52]. Although mechanisms remain under investigation, lncRNAs may influence immune signalling, epithelial–mesenchymal transition, and angiogenesis.

Barriers to clinical translation include inter-study variability, small sample sizes, and non-standardised pre-analytic and analytic protocols, which complicate cross-validation and broader adoption. Most data are retrospective or exploratory; prospective validation in larger, well-defined cohorts is essential. Nevertheless, when integrated with other molecular and clinical data, miRNAs and lncRNAs remain promising components of RCC liquid biopsy, potentially enabling real-time monitoring of tumour evolution, treatment response, and early relapse—particularly in the context of immunotherapy or targeted-therapy resistance. However, most of the available evidence remains exploratory and retrospective, with limited external validation and significant variability in analytical methods.

## 6. KIM-1 as a Tumour and Kidney Injury Biomarker in Renal Cell Carcinoma

Kidney Injury Molecule-1 (KIM-1) is a type I transmembrane glycoprotein with minimal expression in healthy renal parenchyma but marked up-regulation in proximal tubular epithelial cells following injury. Initially identified as a marker of acute and chronic kidney damage, KIM-1 has gained attention in RCC, where it may serve dual functions—as a tumour biomarker and as an indicator of treatment-related nephrotoxicity [53,54]. Importantly, KIM-1 should be considered in two distinct but complementary contexts: as an oncologic biomarker reflecting tumour biology and clinical outcomes and as a marker of kidney injury and treatment-related toxicity.

### 6.1. KIM-1 as an Oncologic Biomarker

In RCC (particularly clear-cell), KIM-1 is aberrantly expressed in tumour tissue. Its soluble ectodomain, shed into circulation, can be measured non-invasively in plasma or urine, supporting incorporation into liquid-biopsy strategies, especially when tissue access is limited or longitudinal monitoring is desired [42,54]. Elevated circulating KIM-1 has been associated with poorer oncologic outcomes: in a prospective nephrectomy cohort, higher postoperative levels independently predicted reduced disease-free and overall survival [55]. Pre-diagnostic plasma KIM-1 has also been correlated with subsequent RCC risk and disease-specific mortality in population-based analyses [56].

Beyond prognostication, KIM-1 may have predictive significance. A post hoc analysis from JAVELIN Renal 101 suggested that baseline KIM-1 levels may be associated with differences in PFS and OS for patients receiving first-line avelumab plus axitinib versus sunitinib [57]; however, these findings remain exploratory and require prospective validation. Although preliminary, this raises the possibility of integrating KIM-1 into baseline risk stratification or early treatment-monitoring frameworks.

### 6.2. KIM-1 as a Marker of Kidney Injury and Treatment-Related Toxicity

In addition, given the nephrotoxic potential of VEGF-targeting TKIs and ICIs, KIM-1 may serve as a sensitive marker of renal epithelial stress or damage [58,59] (Table 2).

Limitations include the absence of standardised assays, heterogeneous cut-offs, and confounding by concurrent non-malignant kidney conditions. Evidence is primarily derived from early-phase and exploratory studies, and prospective validation is required. Until these issues are addressed, KIM-1 should be considered investigational. Ongoing prospective studies are evaluating its integration into prognostic models and surveillance algorithms [42,58]. Overall, KIM-1 is a promising multi-purpose biomarker in RCC, with potential applications spanning diagnosis, prognostication, therapeutic monitoring, and renal safety assessment.

## 7. Angiopoietin-2 as a Circulating Angiogenic Biomarker

Angiopoietin-2 (ANGPT2) is an endothelial-derived mediator that plays a central role in vascular remodelling and angiogenesis and is closely linked to immune modulation within the tumour microenvironment [60,61]. Elevated circulating ANGPT2 levels have been associated with aggressive tumour biology and poorer clinical outcomes across several solid tumours, reflecting vascular instability and a pro-angiogenic, immune-suppressive milieu [1]. In renal cell carcinoma, translational analyses from large, randomised trials suggest that higher baseline ANGPT2 is associated with inferior outcomes and may be associated with reduced benefit from immune checkpoint-based therapies, particularly in tumours characterised by persistent angiogenic signalling [62,63]. Although ANGPT2 is not yet validated as a standalone biomarker in RCC, its role as a predictive biomarker for treatment response remains investigational and requires prospective validation. Nevertheless, its biological relevance and accessibility in blood support its potential incorporation into integrative prognostic models.

## 8. Soluble PD-L1 as a Blood-Based Immune Biomarker

Soluble programmed death-ligand 1 (sPD-L1) represents a circulating form of PD-L1 that reflects systemic immune regulation beyond tumour-cell surface expression. Elevated sPD-L1 levels have been associated with advanced disease stage and poorer survival outcomes in multiple malignancies, including renal cell carcinoma, suggesting a link with immune evasion and global immune suppression [64]. In RCC, circulating sPD-L1 has been correlated with tumour burden and adverse prognosis, although results vary across studies and assay platforms [64]. More recently, PD-L1 has also been detected in urine samples from RCC patients, with levels appearing to correlate with disease activity, supporting a novel, non-invasive liquid biomarker approach [65]. Despite limitations related to assay standardisation and cut-off definitions, sPD-L1 provides complementary biological information to tissue-based PD-L1 assessment and may contribute to multi-parameter immune profiling strategies.

## 9. Conceptual IMDC-Plus Framework for Biologically Informed Risk Re-Stratification

**Figure 1:**
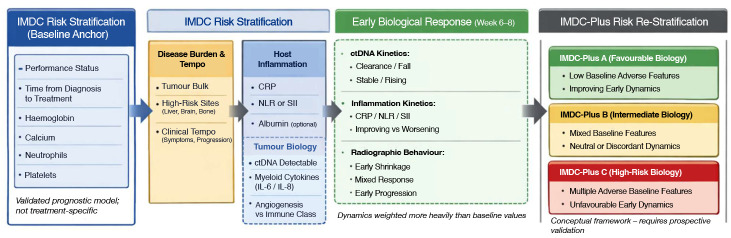
Conceptual IMDC-Plus framework.

With the widespread adoption of immune checkpoint inhibitors in metastatic renal cell carcinoma, the limitations of prognostic models based solely on clinical and laboratory variables have become more evident [1,20]. The IMDC score remains a well-established and prospectively validated tool and continues to provide an important baseline for risk assessment [20]. However, clinical experience and translational studies increasingly suggest that patients within the same IMDC risk category may differ substantially in tumour biology and immune characteristics, which may contribute to heterogeneous treatment outcomes [1,66,67]. To address this gap, conceptual extensions of the IMDC model have been proposed that retain IMDC risk groups as a reference point while incorporating additional biological and clinical context [62,66]. A conceptual overview of this biologically informed IMDC-Plus framework, integrating baseline clinical risk with tumour biology and early on-treatment dynamics, is illustrated in Figure 1.

These include measures of tumour burden and disease kinetics, markers of systemic inflammation, and tumour-specific biological features when available. Such an approach aims to better reflect the underlying biology of the disease rather than relying exclusively on static baseline parameters. An important aspect of this concept is the increased emphasis on early on-treatment dynamics. Changes observed at the first response assessment, typically within the first 6–8 weeks of therapy, may provide more-relevant prognostic information than baseline variables alone [62,63,67]. Early alterations in circulating tumour DNA, trends in inflammatory markers, and initial radiographic behaviour may offer insight into treatment sensitivity and emerging resistance [2,63,68]. Integrating baseline IMDC stratification with early biological and clinical changes may allow more-refined prognostic re-stratification, particularly within the intermediate-risk group, which represents the most heterogeneous IMDC category [1,67]. This IMDC-Plus approach should be viewed as a conceptual model rather than a validated clinical scoring system. Despite its conceptual appeal, several challenges limit the immediate clinical applicability of the IMDC-Plus framework. First, many of the proposed components—such as ctDNA dynamics, circulating biomarkers, and early radiographic changes—lack standardised assays, validated cut-off thresholds, and harmonised reporting methods. Second, integrating multiple data layers into a unified clinical model may increase complexity and limit feasibility in routine practice, particularly in resource-limited settings.

Furthermore, prospective validation of such a model would require biomarker-driven clinical trials with predefined endpoints and longitudinal sampling, which are currently scarce in RCC. The dynamic nature of the proposed framework also raises challenges related to timing of assessments and clinical decision points. Finally, implementation into clinical workflows would require not only analytical validation but also demonstration of clinical utility and cost-effectiveness, which remain to be established.

From a practical standpoint, elements of the IMDC-Plus framework could be implemented in a stepwise manner. In the near term, integration of readily available parameters—such as early changes in inflammatory markers (e.g., CRP and NLR) together with initial imaging response—may represent a feasible approach to dynamic risk re-stratification in routine clinical practice. In contrast, incorporation of more-advanced biomarkers, including circulating tumour DNA, circulating tumour cells, and non-coding RNA assays, is likely to require longer-term development, including assay standardisation, validation in prospective trials, and integration into clinical workflows.

## 10. Future Directions

The landscape of emerging biomarkers in metastatic RCC remains complex and heterogeneous; a summary of key biomarker classes, their potential clinical roles, and current validation gaps are presented in Table 3. Despite rapid advances in biomarker research for RCC, the overall level of clinical evidence remains uneven.

Most candidate biomarkers—including ctDNA metrics, CTC enumeration and characterisation, and circulating non-coding RNAs—are supported primarily by retrospective series or small, single-institution prospective studies. Pre-analytical variability (sample collection, processing, and assay platforms) and the absence of universally accepted cut-off thresholds limit cross-study comparability and delay regulatory acceptance. KIM-1, although promising for both oncologic and nephrologic applications, is similarly confined to exploratory and early validation cohorts. Even the best-established clinical model, the IMDC risk score, functions as a prognostic rather than strictly predictive tool, and its ability to inform the selection of specific ICI or ICI–TKI combinations remains indirect.

From a guideline standpoint, both the European Society for Medical Oncology (ESMO Clinical Practice Guidelines for renal cell carcinoma, 2024) and the National Comprehensive Cancer Network (NCCN Clinical Practice Guidelines in Oncology: Kidney Cancer, Version 2.2025) currently recommend the IMDC model for risk stratification when planning first-line therapy in mRCC, but they do not endorse any molecular or liquid-biopsy biomarker for routine decision-making. These guidelines explicitly describe ctDNA, CTCs, KIM-1, and non-coding RNAs as investigational. Consequently, integration of these emerging biomarkers into everyday clinical algorithms will require large, prospective, biomarker-driven trials and harmonised assay standards before guideline adoption.

Composite multi-omic biomarker panels that combine clinical risk, inflammatory indices, tumour genomics, and liquid-biopsy dynamics are likely to outperform single markers. Harmonised pre-analytics, assay platforms, thresholds, and reporting standards—particularly for ctDNA, CTCs, and non-coding RNAs—are essential for regulatory acceptance. Pragmatic studies embedding biomarker collection into routine workflows will accelerate translation and health-economic evaluation.

## 11. Conclusions

In conclusion, while numerous candidate biomarkers in metastatic RCC show biological and clinical promise, their integration into routine practice remains limited by a lack of prospective validation and standardisation. The future of biomarker-driven care in RCC is unlikely to rely on single markers but rather on integrated, multi-dimensional models that capture tumour biology, host response, and early treatment dynamics. Conceptual frameworks such as the IMDC-Plus model may provide a foundation for this transition; however, their clinical implementation will depend on validation in prospective, biomarker-driven trials and the development of harmonised assay platforms.

## Figures and Tables

**Table 1:** IMDC criteria and risk group definitions.

IMDC Prognostic Factor	Adverse Criterion
Karnofsky performance status	<80%
Time from diagnosis to systemic therapy	<1 year
Haemoglobin	Below lower limit of normal
Corrected calcium	Above upper limit of normal
Absolute neutrophil count	Above upper limit of normal
Platelet count	Above upper limit of normal

**Table 2:** IMDC risk group definitions.

Risk Group	Number of Adverse Criteria	Median Overall Survival (Typical Range)
Favourable	0	~43 months
Intermediate	1–2	~23 months
Poor	≥3	~8 months

Adapted from Heng et al. and subsequent validations [8,9,10,11].

**Table 3:** Emerging biomarkers in metastatic RCC.

Biomarker Type	Representative Examples	Clinical Utility	Current Validation Status/Key Limitations
Clinical/Inflammatory	LDH, CRP, NLR, PLR, IL6, IL8, IL10	Prognostic (OS, PFS); integrated in IMDC	Non-specific; affected by infection/inflammation
Histologic	Sarcomatoid/rhabdoid features	Predictive of ICI benefit; prognostic	Requires expert pathology; intra-tumour heterogeneity
Altered genes	*VHL*, *PBRM1*, *BAP1*, *SETD2*, *MET* (papillary RCC)	Prognostic; potential predictive for ICI or targeted therapy	No standardised testing; prospective trials pending
ctDNA	Mutations in *VHL*, *PBRM1*, *SETD2*; fragmentomics	Monitoring, MRD/early relapse detection, real-time genomics	Low ctDNA abundance; lack of RCC-specific panels
CTCs	Enumeration; PD-L1, MET expression	Prognostic; potential predictive for ICI	Rare cells; no assay standardisation
Non-coding RNAs	miR-210, miR-1233, lncRNA MALAT1	Diagnostic/prognostic; dynamic monitoring	Small cohorts; pre-analytic variability
Protein—KIM-1	Plasma/urine KIM-1	Diagnostic, prognostic, predictive (exploratory), monitoring nephrotoxicity	Assay heterogeneity; confounding kidney disease
Angiogenetic factors	Angiopoietin-2 (Ang-2)	Prognostic; associated with tumour angiogenesis, resistance to VEGF-targeted therapy and inferior outcomes with ICI	Not standardised; variable cut-offs; mainly exploratory/retrospective data
Soluble immune checkpoints	Soluble PD-L1 (sPD-L1)	Prognostic; potential predictive biomarker for ICI response; dynamic monitoring during treatment	Assay variability; lack of validated thresholds; limited prospective validation

## Data Availability

Not applicable.
